# Bacterial Vaginosis Biofilms: Challenges to Current Therapies and Emerging Solutions

**DOI:** 10.3389/fmicb.2015.01528

**Published:** 2016-01-20

**Authors:** Daniela Machado, Joana Castro, Ana Palmeira-de-Oliveira, José Martinez-de-Oliveira, Nuno Cerca

**Affiliations:** ^1^Laboratory of Research in Biofilms Rosário Oliveira, Centre of Biological Engineering, University of MinhoBraga, Portugal; ^2^Instituto de Ciências Biomédicas Abel Salazar, Universidade do PortoPorto, Portugal; ^3^Health Sciences Research Center, Faculty of Health Sciences, University of Beira InteriorCovilhã, Portugal; ^4^Labfit, Health Products Research and Development LdaCovilhã, Portugal; ^5^Child and Woman's Health Department, Centro Hospitalar Cova da BeiraCovilhã, Portugal

**Keywords:** bacterial vaginosis, biofilms, *Gardnerella vaginalis*, antibiotics, emerging therapies

## Abstract

Bacterial vaginosis (BV) is the most common genital tract infection in women during their reproductive years and it has been associated with serious health complications, such as preterm delivery and acquisition or transmission of several sexually transmitted agents. BV is characterized by a reduction of beneficial lactobacilli and a significant increase in number of anaerobic bacteria, including *Gardnerella vaginalis, Atopobium vaginae, Mobiluncus* spp., *Bacteroides* spp. and *Prevotella* spp.. Being polymicrobial in nature, BV etiology remains unclear. However, it is certain that BV involves the presence of a thick vaginal multi-species biofilm, where *G. vaginalis* is the predominant species. Similar to what happens in many other biofilm-related infections, standard antibiotics, like metronidazole, are unable to fully eradicate the vaginal biofilm, which can explain the high recurrence rates of BV. Furthermore, antibiotic therapy can also cause a negative impact on the healthy vaginal microflora. These issues sparked the interest in developing alternative therapeutic strategies. This review provides a quick synopsis of the currently approved and available antibiotics for BV treatment while presenting an overview of novel strategies that are being explored for the treatment of this disorder, with special focus on natural compounds that are able to overcome biofilm-associated antibiotic resistance.

## Introduction

The healthy vaginal microflora has been described as being constituted mainly by Gram-positive bacilli of the genus *Lactobacillus*, being *L. crispatus, L. iners, L. gasseri*, and *L. jensenii* the most common species (Ravel et al., 2011). However, other non-beneficial microbial species, including *Gardnerella vaginalis, Enterococcus spp*., and *Prevotella spp.*, can be present in small numbers, not sufficient to cause disease (Marrazzo et al., 2002). Importantly, lactobacilli play a pivotal role in maintaining the female genital tract health while preventing genitourinary infections (Borges et al., 2014).

Among genital infections, bacterial vaginosis (BV) is the leading vaginal disorder in women of childbearing age, contributing to more than 60% of all vulvovaginal infections (Sobel, 2000). BV, as a whole, has been associated with serious health problems, including pre-term birth (Leitich et al., 2003), spontaneous abortion (Guerra et al., 2006), pelvic inflammatory disease (Rothman et al., 2003), endometritis (Jacobsson et al., 2002) and acquisition and transmission of several sexually transmitted agents (Gallo et al., 2012). Clinically, a profuse vaginal discharge and a rotten fish vaginal odor are characteristic symptoms, although some women with BV remain asymptomatic (Koumans et al., 2007). Microbiologically, this condition is characterized by a dramatic shift of vaginal microflora which involves the loss of beneficial bacteria (lactobacilli) and a simultaneous proliferation of anaerobic bacteria including *G. vaginalis, Atopobium vaginae, Mobiluncus* spp., *Bacteroides* spp., and *Prevotella* spp. (Verhelst et al., 2004). Its high prevalence and the associated complications make BV an important public health issue. However, due to the great diversity and complexity of microorganisms involved, the BV etiopathogenesis is not yet fully understood and is still a matter of controversy (Schwebke et al., 2014).

Back in 1955, Gardner and Dukes proposed that *G. vaginalis* was the sole etiological agent of BV (Gardner and Dukes, 1955). However, their findings were disputed when some years later *G. vaginalis* was found in approximately 40% of healthy women. In addition, other anaerobic bacteria were positively associated with BV and this lead researchers to conclude that BV was a polymicrobial infection (Sobel, 2000). However, a major conceptual problem with this later hypothesis is its inconsistency with epidemiological data, which suggests that BV is a sexual transmitted disease and therefore, most likely to be caused by a single agent (Josey and Schwebke, 2008). Currently, is consensual that BV involves the presence of a dense, structured and polymicrobial biofilm, primarily constituted by *G. vaginalis* clusters, strongly adhered to the vaginal epithelium (Swidsinski et al., 2005). Biofilms are communities of microorganisms attached to a surface and encased in a polymeric matrix of polysaccharides, proteins and nucleic acids (Høiby et al., 2011). Due to the fact that bacteria within biofilms are not effectively eliminated by the immune system (Cerca et al., 2006; Xie et al., 2012) or fully destroyed by antibiotics (Cerca et al., 2005; Tobudic et al., 2012), biofilm-related infections tend to persist and so, not surprisingly, BV tends to have a high rate of relapse and recurrence (Bradshaw et al., 2006). So, the current paradigm is that the establishment of a *G. vaginalis* biofilm is a required event for initiation and progression of BV (Machado and Cerca, 2015). In fact, *in vitro* studies demonstrated that *G. vaginalis* biofilm displays a high resistance to the protective mechanisms of normal vaginal microflora, including hydrogen peroxide, and lactic acid produced by lactobacilli (Patterson et al., 2007), as well as an increased tolerance to antibiotics (Swidsinski et al., 2008). Therefore, vaginal biofilms play a key role not only in BV pathogenesis, but also in its treatment failure and recurrence. Thus, the purpose of this review is to present currently approved and available therapeutic strategies for BV, as well as to discuss the emerging therapies that are being explored for BV treatment, attributing more emphasis to novel therapeutics targeting vaginal biofilms.

## Current BV antibiotic therapeutic options

Despite the most recent discoveries related to the etiology of BV, current treatment is still directed toward alleviation of symptoms through reduction of BV-associated bacteria overgrowth and restoration of normal vaginal flora (Pirotta et al., 2009). The Centers for Disease Control and Prevention (CDC) recommends that all symptomatic women should be treated, since it recognizes several benefits of therapy, including the relief of the symptoms and signs of infection (Centers for Disease Control and Prevention, 2015) and the reduction in the risk of acquiring sexually transmitted diseases (Brotman et al., 2010). Conventionally, BV is treated with metronidazole, clindamycin or tinidazole (Centers for Disease Control and Prevention, 2015).

Presently, metronidazole is considered to be the drug of choice for BV treatment (Centers for Disease Control and Prevention, 2015). It is a first generation nitroimidazole, which was initially indicated for the management of trichomoniasis (Moffett and Mcgill, 1960) but was then shown to be effective against anaerobic microorganisms (Tally et al., 1975). However, metronidazole therapy is associated with several side effects such as nausea, vomiting and gastrointestinal complaints (Schwebke and Desmond, 2011; Abdali et al., 2015; Chavoustie et al., 2015; Schwebke et al., 2015). Clindamycin is also an antimicrobial agent for BV treatment (Centers for Disease Control and Prevention, 2015), with similar efficacy as metronidazole (Paavonen et al., 2000; Beigi et al., 2004). It is a lincosamide that is available in various pharmaceutical formulations including vaginal dosage forms and oral (systemic) pills(Menard, 2011). However, when applied topically, clindamycin might weaken latex products such as condoms (Rosen and Rosen, 1999) and may even cause pseudomembranous colitis (Trexler et al., 1997). Tinidazole was the most recently approved antimicrobial agent for BV treatment, by the Food and Drug Administration (Dickey et al., 2009) and it is considered an alternative antimicrobial agent for BV treatment particularly whenever metronidazole and clindamycin are unavailable or not tolerated (Centers for Disease Control and Prevention, 2015). Being a second generation nitroimidazole with a longer half-life than metronidazole (Wood and Monro, 1975) it requires lower dosages, to be taken less frequently than metronidazole (Dickey et al., 2009). Other antibiotics like ornidazole (Thulkar et al., 2012), secnidazole (Núñez and Gómez, 2005; Bohbot et al., 2010; Thulkar et al., 2012) and azithromycin (Nikolov et al., 2008) have been tested as alternatives to treat BV, however these antibiotics are not currently approved by the Food and Drug Administration and have not shown to increase BV cure rates.

Despite the high cure rates achieved in some studies, very high BV recurrence rates and some relevant treatment side effects have been reported. A list of the most recent studies (2010–2015) is presented in Table 1.

**Table 1:** **Studies of common antibiotics used in BV treatment, published between 2010 and 2015**.

**Antibiotic**	**Regimen**	**Treatment cure rate (*n* = population size)**	**Recurrence rate (*n* = population size)**	**Side effects**	**References**
Clindamycin	300 mg of oral clindamycin (2 × D for 1 W)	84% (*n* = 150)	6% (*n* = 150)	Preterm labor (4.7%); premature rupture of the membranes (3.3%)	Hantoushzadeh et al., 2012
Metronidazole	250 mg of oral metronidazole (2 × D for 1 W)	97.1% (*n* = 70)	N/A	Metallic taste (34.3%); nausea (21.4%); dizziness (11.4%); vomiting (4.3%)	Abdali et al., 2015
Metronidazole	500 mg of oral metronidazole (2 × D for 1 W)	48.3% (*n* = 60)	N/A	Heartburn (15%); metallic taste (11.7%); headache (6.7%); skin rash (1.7%); vomiting (1.7%); diarrhea (1.7%)	Mohammadzadeh et al., 2014
Metronidazole	750 mg of intravaginal metronidazole plus 200 mg of intravaginal miconazole (2 × W for 3 M)	100% (*n* = 16)	66.7% (*n* = 9) post-BV treatment	N/D	Aguin et al., 2014
Metronidazole	500 mg of oral metronidazole (2 × D for 1 W) and topical metronidazole cream (for 5 D)	N/A	>50% after 12 M post-BV treatment	N/A	Bodean et al., 2013
Metronidazole	0.8% metronidazole *in situ* gel (2 × D for 5 D)	85% (*n* = 20)	N/A	N/A	Shaaban et al., 2011
Metronidazole	0.8% metronidazole gel (2 × D for 5 D)	71.4% (*n* = 21)	N/A	N/A	Shaaban et al., 2011
Metronidazole	500 mg of oral metronidazole (2 × D for 1 W)	82.4% (*n* = 136)	33.3% (*n* = 102) after 1 M and 33.9% (*n* = 56) after 2 M post-BV treatment	Yeast infection (29.3%); nausea/vomiting (20.2%); headache (14.7%); bad taste (11%); diarrhea (3.7%); anorexia (0.8%)	Schwebke and Desmond, 2011
Metronidazole	1.3% metronidazole gel (1 × D for 1 D)	30.2% (*n* = 43)	52% (*n* = 25) of abnormal discharge and fishy odor post-BV treatment	Vulvovaginal candidiasis (12.3%); headache (4.6%); nasopharyngitis (3.1%); vulvovaginal pruritus (3.1%); nausea (1.5%)	Chavoustie et al., 2015
Metronidazole	1.3% metronidazole gel (1 × D for 3 D)	25% (*n* = 48)	58.6% (*n* = 29) of abnormal discharge and fishy odor post-BV treatment	Vulvovaginal candidiasis (13.3%); headache (8.3%); vulvovaginal pruritus (6.7%); nasopharyngitis (5%)	Chavoustie et al., 2015
Metronidazole	1.3% metronidazole gel (1 × D for 5 D)	32.7% (*n* = 49)	21.4% (*n* = 28) of abnormal discharge and fishy odor post-BV treatment	Headache (6.3%); nausea (6.3%); nasopharyngitis (1.6%)	Chavoustie et al., 2015
Metronidazole	0.75% metronidazole gel (1 × D for 5 D)	20.4% (*n* = 49)	50% (*n* = 26) of abnormal discharge and fishy odor post-BV treatment	Vulvovaginal candidiasis (13.8%); headache (13.8%); nasopharyngitis (1.5%); vulvovaginal pruritus (1.5%)	Chavoustie et al., 2015
Metronidazole	1.3% metronidazole gel (1 × D for 1 D)	37.2% (*n* = 250)	N/A	Vulvovaginal infections (5.6%); headache (2.2%); nausea (1.6%); vulvovaginal pruritus (1.6%); diarrhea (1.2%); dysmenorrhea (1.2%)	Schwebke et al., 2015
Metronidazole	500 mg of intravaginal metronidazole (1 × D for 1 W)	83.3% (*n* = 30)	20% (*n* = 25) after 1 M post-BV treatment	N/A	Ling et al., 2013
Metronidazole	2 g of oral metronidazole in (1 × D for 1 D)	88.4% (*n* = 86)	N/A	N/A	Thulkar et al., 2012
Metronidazole	0.75% metronidazole gel (2 × D for 5 D)	71.3% (*n* = 108)	N/A	Adverse events in 15.6% of patients	Zeng et al., 2010
Ornidazole	1.5 g of oral ornidazole in (1 × D for 1 D)	100% (*n* = 86)	N/A	N/A	Thulkar et al., 2012
Rifaximin	100 mg of vaginal rifaximin (for 5 D)	25.9% (n = 27)	N/A	Vulvovaginal candidiasis (7.4%); vulvovaginal pruritus (3.7%); vaginal inflammation (3.7%)	Donders et al., 2013
Rifaximin	25 mg of vaginal rifaximin (for 5 D)	48% (*n* = 25)	N/A	Vulvovaginal candidiasis (16%); diarrhea (8%); gastrointestinal symptoms and headache (4%); vulvovaginal discomfort (4%)	Donders et al., 2013
Rifaximin	100 mg of vaginal rifaximin (for 2 D)	36% (*n* = 25)	N/A	Vulvovaginal candidiasis (8%)	Donders et al., 2013
Secnidazole	2 g of oral secnidazole in (1 × D for 1 D)	90.7% (*n* = 86)	N/A	N/A	Thulkar et al., 2012
Secnidazole	2 g of oral secnidazole (1 × D for 1 D)	62.4 % (*n* = 290)	N/A	Headache (9%)	Bohbot et al., 2010
Tinidazole	500 mg of oral tinidazole (2 × D for 1 W)	75.3% (*n* = 146)	30.2% (*n* = 96) after 1 M and 40% (*n* = 55) after 2 M post-BV treatment	Yeast infection (25%); nausea/vomiting (19.7%); headache (16.7%); bad taste (15.2%); diarrhea (0.8%); anorexia (5.3%)	Schwebke and Desmond, 2011
Tinidazole	1 g of oral tinidazole (2 × D for 1 W)	73% (*n* = 137)	22.5% (*n* = 89) after 1M and 20.3% (*n* = 59) after 2M post-BV treatment	Bad taste (41.8%); nausea/vomiting (30.9%); headache (24.5%); yeast infection (24.5%); diarrhea (5.5%); anorexia (4.5%)	Schwebke and Desmond, 2011
Tinidazole	2 g of oral tinidazole in (1 × D for 1 D)	100% (*n* = 86)	N/A	N/A	Thulkar et al., 2012

(D), Daily or day; (W), Weekly or Weeks; (M), Monthly or Months; (N/A)–in that study, this was not quantified; (N/D)–in that study, adverse effects were non-detected.

The low efficacy of antibiotics in preventing recurrences is thought to be due to their inability to fully eradicate BV vaginal biofilms-associated bacteria. In fact, Swidsinski and colleagues investigated the influence of oral metronidazole therapy on *G. vaginalis* biofilms and reported that biofilms were only temporarily suppressed, and that in most cases rapidly regained activity following treatment cessation (Swidsinski et al., 2008). Later, Alves and colleagues determined the *in vitro* susceptibility of 30 BV-associated biofilm forming bacteria to metronidazole, tinidazole, and clindamycin and showed that all tested bacteria tested were resistant to metronidazole and tinidazole and 67% to clindamycin (Alves et al., 2014). In this sense, novel antimicrobials agents, with the ability to selectively target vaginal pathogens and their biofilms, are urgently required.

## Emerging therapeutic alternatives against BV

The increasing evidence that BV is a biofilm-mediated infection sparked the interest of the scientific community in exploring agents aimed to disrupting biofilms. Thus, in recent years, studies of anti-BV agents started to include biofilm disruptor candidates, such as DNases, retrocyclins, probiotics, antiseptics, natural antimicrobials, and plant-derived compounds (see Table 2).

**Table 2:** **Emerging (2013–2015) therapeutic strategies against BV-related vaginal biofilms**.

**Agent**	**Application/Tested in (*n* = population size, in *in vivo* studies)**	**Main results**	**References**
**PLANTS AND PLANTS EXTRACTS**			
***In vivo***			
Extract of the Brazilian pepper tree	Evaluate the effect of gel containing 7.4% extract of the Brazilian pepper tree (1 D × 1 W); *n* = 137 BV women	Low cure rate (13.9%). Side effects including abdominal pain; heat; burning; rash	Leite et al., 2011
Garlic tablet	Analyze the effect of oral garlic (2 × D for 1 W); *n* = 60 BV women	Moderate cure rate (63.3%). Side effects (15%)	Mohammadzadeh et al., 2014
*Zataria multiflora*	Analyze the efficiency of 5 g of intravaginal cream of 0.1% *Zataria multiflora* (for 7 D) *n* = 70 BV women	High cure rate (92.9%). Alleviation of BV-symptoms	Abdali et al., 2015
***In vitro***			
19 plant extracts tested	Test the antimicrobial activity of extracts in *G. vaginalis*	7 plant extracts showed antimicrobial activity	Naidoo et al., 2013
Seaweed extracts	Screening involving 44 species of seaweed extract with potential anti-*G. vaginalis* activity	Extracts of the seaweed *Ulva pertusa* displayed a strongest activities against *G. vaginalis*	Ha et al., 2014
**PROBIOTICS**			
***In vivo***			
*L. crispatus* DM8909	Evaluate the efficacy of intravaginal capsule of probiotics (1 × D for 10 D) *n* = 25 BV women	High cure rate (96%) 30 D after beginning the BV treatment	Ling et al., 2013
*L. fermentum* LF15 and *L. plantarum* LP01	Evaluate the efficacy of probiotics to improve the Nugent score *n* = 24 BV women	Lactobacilli significantly reduced the Nugent score below the threshold of 7 after 28 D	Vicariotto et al., 2014
*L. rhamnosus* GR-1 and *L. reuteri* RC-14	Evaluate the effect of oral capsules of probiotics (1 × D for 6 W) *n* = 395 BV women	Normal vaginal microbiota were present in more than half of patients (51.1%). Undisclosed side effects reported	Vujic et al., 2013
VSL3^®^	Evaluate the efficacy of oral probiotic (2 × D for 5 D, followed by 1 × D for 10 D) *n* = 20 pregnant women with BV	Absent of vaginal discharge; reduction of the itching and leukorrhea; improvement of constipation occurs	Facchinetti et al., 2013
***In vitro***			
*L. fermentum* LF15	Analyze the inhibitory activity of the neutralized supernatants of probiotic against *G. vaginalis*	Reduced the Nugent score below the threshold of 7. *L. fermentum* LF15 showed an inhibitory activity against *G. vaginalis*	Vicariotto et al., 2014
*L. fermentum* L23	Analyze the colonization ability and curative effect of probiotic in female BALB/c mice infected with *G. vaginalis*.	*L. fermentum* L23 inhibited the growth of *G. vaginalis*	Daniele et al., 2014
*L. fermentum* SK5	Analyze the effect of probiotic in adhesion of *E. coli* and *G. vaginalis* to intestinal and vaginal cells, respectively	*L. fermentum* SK5 inhibit pathogenic microorganisms by production antimicrobial substance (bacteriocin-like and hydrogen peroxide)	Kaewnopparat et al., 2013
**ANTIMICROBIAL PEPTIDES**			
***In vitro***			
Fermenticin HV6b	Evaluate the antimicrobial activity of bacteriocin in *G. vaginalis*	Inhibition of *G. vaginalis*; immobilization and spermicidal activity; induce apoptosis in cancerous cells	Kaur et al., 2013
Retrocyclin	Test the effect of retrocyclin in BV-associated bacteria	Pathogenic vaginal bacteria were inhibited by retrocyclin. Retrocyclin was well-tolerated by host tissues and by commensal vaginal bacteria	Eade et al., 2013
**ADJUVANTS OF ANTIBIOTIC THERAPHY**			
***In vivo***			
EcoVag® with clindamycin or metronidazole	Analyze the efficiency of 2% clindamycin cream (1 × D for 1 W), 300 mg of oral clindamycin (for 1W) and oral probiotic capsules (for 5D). After the next menstruation was applied 0.75% of metronidazole gel (5D) and probiotic capsules (for 5D) *n* = 10 BV women	Treatment with antibiotics in combination with EcoVag® provide long-term cure against BV	Pendharkar et al., 2015
*L. acidophilus* and *L. bifidus* with metronidazole	Analyze the efficiency of 500 mg of oral metronidazole (2 × D for 1 W), metronidazole cream (for 5 D) and oral probiotic capsules (2 × D for 10 D) *n* = 173 BV women	Low recurrence rate (15%) after 3 M post-BV treatment. Undisclosed side effects reported	Bodean et al., 2013
*L. acidophilus* and *L. rhamnosus* with tinidazole and metronidazole	Analyze the efficiency of 2 g of tinidazole (for 2 D), vaginal suppositories of 1 g of metronidazole (D1 and D3) and topical vaginal probiotics (from the 5 D of the treatment) *n* = 297 BV women	Reduction of BV recurrence. Recolonization of the vagina with lactobacilli	Kovachev and Vatcheva-Dobrevski, 2013
*L. casei var rhamnosus* - Lcr 35 with metronidazole	Evaluate the efficiency of 500 mg of metronidazole (2 × D for 5 D), local application of 1 g of metronidazole ovules (D1 and D3) and vaginal ovules of probiotic (2 × D for 7 D) *n* = 30 BV women	Increased the clinical and microbiological efficacy of the antibiotic therapy and restore the microbial balance in the vaginal ecosystem	Kovachev and Dobrevski-Vacheva, 2013
*L. rhamnosus* BMX 54 with metronidazole	Evaluate the efficiency of 500 mg of oral metronidazole and probiotic (2 × D for 1 W) *n* = 125 BV women	Significant replace of the BV-associated flora by a health vaginal flora and re-establishment of the physiological acid vaginal after 2 M of treatment	Recine et al., 2016
*L. rhamnosus, L. acidophilus, S. thermophilus* and *L. bulgaricus* with metronidazole	Evaluate the efficiency of 500 mg of oral metronidazole (2 × D for 7 D), topical metronidazole cream (for 5 D) and vaginal ovules of probiotics (1 × D for 6 D) *n* = 173 BV women	Recurrence rate was 30%. Undisclosed side effects reported	Bodean et al., 2013
Miconazole with metronidazole	Evaluate the efficiency of 750 mg of vaginal suppositories of metronidazole and 200 mg of miconazole (5 consecutive D for each M for 12 M) *n* = 116 BV women	Monthly treatment with intravaginal metronidazole plus miconazole reduced the proportion of visits with BV during 12 M of follow-up	McClelland et al., 2015
Vitamin C with metronidazole or clindamycin	Evaluate the efficiency of 250 mg of vitamin C as prophylaxis (6 D each M for 6 M) after episode of BV treated either metronidazole or clindamycin *n* = 74 BV women	Reduction of BV recurrence (32.4% to 16.2%). Side effects including burning, itching, skin irritation, candidiasis and bronchitis	Krasnopolsky et al., 2013
Vitamin D with metronidazole	Evaluate the efficiency of 500 mg of oral metronidazole (2 × D for 7 D) and 9 doses of vitamin D (for 24 W) *n* = 59 women	BV recurrence was not reduced by vitamin D supplementation	Turner et al., 2014
***In vitro***			
Lauramide arginine ethyl ester with clindamycin	Evaluate the impact of this therapy in bacterial biofilms of *Lactobacillus* spp., *G. vaginalis* and other BV-associated bacteria	LAE synergized with clindamycin against biofilms of *G. vaginalis* but not biofilm-associated vaginal lactobacilli	Algburi et al., 2015
Lauramide arginine ethyl ester with metronidazole	Evaluate the impact of this therapy in bacterial biofilms of *Lactobacillus* spp., *G. vaginalis* and other BV-associated bacteria	LAE synergized with metronidazole against biofilms of *G. vaginalis* but not biofilm-associated vaginal lactobacilli	Algburi et al., 2015
Subtilosin with clindamycin	Evaluate the impact of this therapy in bacterial biofilms of *Lactobacillus* spp., *G. vaginalis* and other BV-associated bacteria	Subtilosin synergized with clindamycin against biofilms of *G. vaginalis* but not biofilm-associated vaginal lactobacilli	Algburi et al., 2015
Subtilosin with clindamycin	Evaluate the synergistic potential of two-antimicrobial combinations against *G. vaginalis* and *Lactobacillus* spp.	Synergistic effect against *G. vaginalis* in terms of fractional inhibitory concentration index (FICI)	Cavera et al., 2015
Subtilosin with metrodinazole	Evaluate the impact of this therapy in bacterial biofilms of *Lactobacillus* spp., *G. vaginalis* and other BV-associated bacteria	Subtilosin synergized with metronidazole against biofilms of *G. vaginalis* but not biofilm-associated vaginal lactobacilli	Algburi et al., 2015
Subtilosin with metrodinazole	Evaluate the synergistic potential of two-antimicrobial combinations against *G. vaginalis* and *Lactobacillus* spp.	Synergistic effect against *G. vaginalis* in terms of fractional inhibitory concentration index (FICI)	Cavera et al., 2015
**OTHERS**			
***In vivo***			
Vitamin C	Evaluate the efficiency of drops of 250 mg of vaginal tablets of vitamin C (1 × D for 6 D, followed by 1 × W for 12 W) *n* = 70 women with abnormal microflora which 16 women had BV	Vaginal ascorbic acid improves abnormal vaginal pH and microflora, especially in pregnant women, but is not well tolerated by all women	Zodzika et al., 2013
Vitamin D	Evaluate the efficiency of drops of vitamin D (1 × D for 15W) n = 105 BV women	Moderate cure rate (63.5%). The administration of 2000 IU/day edible vitamin D was effective in eliminating asymptomatic BV	Taheri et al., 2015
Octenidine dihydrochloride spray application	Evaluate the efficiency of octenidine (for 7 D) *n* = 24 women with recurrent BV	High initial cure rate (87.5%, after 10 D of treatment). Moderate cure rate after 12 M post-BV treatment (62.5%). A complete resistance to octenidine was verified in some of patients after 1 year of treatment (37.5%)	Swidsinski et al., 2015
Estriol vaginal tablets and prebiotic lactoferrin (LF)	Evaluate the efficiency of 150 mg of estriol vaginal tablets and 700 mg LF (1 × D) *n* = 1 BV pregnant women	Recolonization of the vagina with lactobacilli was detected after 1 M to start the treatment. There were no findings of fetal disorders and placental abnormalities	Otsuki et al., 2014
***In vitro***			
Benzoyl Peroxide formulated Polycarbophil/Carbopol 934P Hydrogel	Evaluate the antimicrobial activity of benzoyl peroxide encapsulated in a hydrogel against *G. vaginalis* and *Lactobacillus* spp.	Inhibition the growth of *G. vaginalis*. Limited effect on healthy lactobacilli in the vaginal ecosystem	Xu et al., 2013
DNAses	Evaluate the effect of DNAses in *G. vaginalis* biofilms and in a murine vaginal colonization model	50% of biofilm inhibition at 100 μg/mL DNase and >10-fold inhibition of *G. vaginalis* colonization by DNase in a murine vaginal colonization model	Hymes et al., 2013
Subtilosin within covalently cross-linked polyethylene glycol (PEG)-based hydrogels	Effect of subtilosin within covalently cross-linked polyethylene glycol (PEG)-based hydrogels on *G. vaginalis* and *Lactobacillus* spp.	The subtilosin-containing hydrogels inhibited the growth of *G. vaginalis*. The growth of vaginal lactobacilli was not significantly inhibited	Sundara Rajan et al., 2014

(D), Daily or day; (W), Weekly or Weeks; (M), Monthly or Months.

### Antiseptics

During several decades, antiseptics have been applied in the management of vaginal infections (Ratzan, 1969; Ison et al., 1987). They have an antibacterial activity against a broad spectrum of bacteria, acting by nonspecifically disrupting their cell membrane (Lachapelle et al., 2013). A great panoply of antiseptics have been used to treat BV, including dequalinium chloride (Petersen et al., 2002), povidone iodide (Wewalka et al., 2002), hydrogen peroxide (Cardone et al., 2003), polyhexamethylene biguanide (Gerli et al., 2003), chlorhexidine (Molteni et al., 2004), octenidine hydrochloride/phenoxyethanol (Novakov Mikic and Budakov, 2010), nifuratel (Togni et al., 2011), and benzydamine hydrochloride (Boselli et al., 2012). However, a recent systematic review verified that most studies addressing the use of antiseptics for BV treatment are somehow methodologically weak since follow-up studies were very limited and their safety and excipients composition was poorly investigated (Verstraelen et al., 2012). Nevertheless, the potential of antiseptics against BV biofilms was recently highlighted when Swidsinski and colleagues reported high initial cure rates when using octenidine. However, the efficacy of prolonged and repeated treatment was lower than expected and bacterial resistance emerged in a considerable subset of women (Swidsinski et al., 2015).

### Probiotics and prebiotics

An alternative approach to deal with BV is by modulating the vaginal microbiota, for example, by using probiotics. Probiotics are live microorganisms which confer a health benefit to the host, when administered in suitable amounts (Food Agriculture Organization of the United Nations World Health Organization, 2001). In the human vagina, certain *Lactobacillus* strains can act as probiotics, preventing the growth of BV-associated bacteria through two main mechanisms: the inhibition of pathogens adhesion to vaginal epithelium (Machado et al., 2013); and the production of antimicrobial compounds like hydrogen peroxide (Mastromarino et al., 2002), lactic acid (Boskey et al., 2001) and bacteriocins (Aroutcheva et al., 2001b). Diverse pharmaceutical formulations containing probiotic lactobacilli strains have reduced BV symptoms, improved the vaginal microflora profile, being usually well-tolerated (Rossi et al., 2010; Hantoushzadeh et al., 2012; Facchinetti et al., 2013; Vujic et al., 2013; Vicariotto et al., 2014). In contrast, despite their therapeutic potential, some clinical trials have not detected a significant improvement in BV management (Falagas et al., 2007). Alternatively, probiotics have been proposed as adjuvants to antibiotic therapy. Several combinations of metronidazole, clindamycin or tinidazole with lactobacilli probiotic preparations have displayed promising results in BV treatment since they have been associated with high cure rates, low recurrence or quick re-establishment of an healthy vaginal microflora (Marcone et al., 2010; Bodean et al., 2013; Recine et al., 2016). Probiotics have also been used in an attempt to specifically deal with BV biofilms. Remarkably, in 2007, Saunders and colleagues showed that L. *reuteri* RC-14 was able to disrupt *in vitro G. vaginalis* biofilms (Saunders et al., 2007). Later, McMillan and colleagues demonstrated that probiotic *L. reuteri* RC-14 and *L. rhamnosus* GR-1 were able to incorporate themselves into BV-biofilm, composed by *G. vaginalis* and *A. vaginae*, causing both the disruption of the biofilm structure and bacterial cell death (McMillan et al., 2011). These findings provide some evidence of how lactobacilli probiotics might interfere with an abnormal vaginal microflora, reinforcing the hypothesis that probiotics could eradicate vaginal pathogenic biofilms and restore the normal microflora in *in vivo* situations.

It has also been proposed that prebiotics, nutritional substances that stimulate the growth of probiotics, could be used as alternative to treat BV (Roberfroid, 2007). Interestingly, Rousseau and colleagues demonstrated that prebiotic preparations containing oligosaccharides were able to promote the growth of beneficial lactobacilli strains but not of the pathogenic microorganisms often found in urogenital infections including *G. vaginalis* (Rousseau et al., 2005). Later, Zeng and colleagues compared the efficacy of a prebiotic gel containing sucrose with 0.75% metronidazole vaginal gel to treat BV (Zeng et al., 2010). In that study, the prebiotic gel displayed a similar therapeutic cure rate to metronidazole, having a major advantage of quicker restoration of the normal vaginal microflora. Recently, Coste and colleagues evaluated the efficacy and safety of another prebiotic gel, applied as adjuvant therapy, in women treated for BV and showed an improved recovery of the normal vaginal flora, reducing the risk of recurrences (Coste et al., 2012).

### Plant-derived compounds

The use of plant-derived compounds in the treatment of genital infections is another therapy on the rise (Palmeira-de-Oliveira et al., 2013). One of the earliest reports on this topic dates back from 1991, when Blackwell described the first therapeutic success of using plants extracts to treat BV (Blackwell, 1991). Subsequently, several clinical trials have demonstrated that the use of plant-derived compounds promoted the reduction of BV symptoms and are associated with high cure rates and tolerability, including a polyherbal vaginal pessary (Patel et al., 2008), vaginal cream containing *Zataria multiflora* (Abdali et al., 2015), a vaginal douche of thymol and eugenol (main constituents of thyme oil and clove oil; Sosto et al., 2011), watery extract of *Triticum vulgare* (Boselli et al., 2012) and garlic tablets (Mohammadzadeh et al., 2014). Surprisingly, up to now only one study evaluated the capability of plant-derived compound to eradicate BV biofilms. Interestingly, Braga and colleagues showed that thymol, a molecule present in thyme essential oil, had an inhibitory effect upon both newly formed and mature *G. vaginalis* biofilms, which supports the importance of exploring essential oils and their main constituents as therapeutic alternative to treat BV (Braga et al., 2010). Furthermore, the expectations on essential oils as effective agents against BV-biofilms can be inferred from studies in other related vaginal biofilms (Palmeira-de-Oliveira et al., 2012; Bogavac et al., 2015).

### Natural antimicrobials

Natural antimicrobials, mainly bacteriocins, have also been studied as potential therapeutic alternatives against BV. Several natural antimicrobials, including *L. acidophilus* 160 bacteriocin (Aroutcheva et al., 2001a), subtilosin (Sutyak et al., 2008, 2012; Cavera et al., 2015), lactocin 160 (Turovskiy et al., 2009), lactosporin (Riazi et al., 2012), fermenticin HV6b (Kaur et al., 2013), polylysine (Sutyak et al., 2012; Cavera et al., 2015), lauramide arginine ethyl ester (LAE) (Cavera et al., 2015; Sutyak et al., 2012) and glycerol monolaurate (Strandberg et al., 2010; Sutyak et al., 2012), displayed an inhibitory effect against BV-associated bacteria grown planktonically, usually not affecting the lactobacillary flora. Due to this important advantage, natural antimicrobials have also been proposed as a valuable therapeutic alternative to eradicate BV-biofilms. Remarkably, Turovskiy and colleagues tested the susceptibility of *G. vaginalis* biofilms to several natural antimicrobials. Using a series of *in vitro* assays, these researchers demonstrated that LAE had the strongest bactericidal effect against *G. vaginalis* biofilms, proposing LAE as a potential natural agent to disrupt BV-biofilm (Turovskiy et al., 2012). Later, Algburi and colleagues showed that subtilosin and LAE showed synergistic effect with clindamycin and metronidazole, inhibiting *G. vaginalis* biofilms, while not disturbing vaginal lactobacilli (Algburi et al., 2015). This demonstrated that the combination of conventional antibiotics with natural antimicrobials can improve the cure rates of antibiotic therapy, especially in cases where antimicrobial resistant was found.

### Acidifying/buffering agents

Another interesting approach to treat BV is vaginal acidification (Boskey et al., 1999). However, the results concerning this strategy are controversial since acidification strategies alone, using acetic acid (Holley et al., 2004) or acid-buffering formulation (Simoes et al., 2006) showed to be somewhat ineffective against BV. Recently, Bahamondes and colleagues verified that a soap containing lactic acid and lactoserum could be used for external intimate hygiene, reducing BV recurrence after treatment with oral metronidazole (Bahamondes et al., 2011). Interestingly, vitamin C, when coated with silicon, allowed the constant release of the active agent, resulting in a long-lasting vaginal low pH and prevention of vaginal irritation (Polatti et al., 2006). Other studies reported an effective and safe use of vaginal vitamin C tablets in BV treatment (Petersen et al., 2011), contributing to improve abnormal vaginal pH and microflora, especially in pregnant women (Zodzika et al., 2013). Additionally, the regular use of vitamin C during 6 days per month, for 6 months after successful BV treatment, was shown to decrease the risk of BV recurrence (Krasnopolsky et al., 2013). Another alternative comes in the form of buffering agents. Polycarbophil is a weak poly-acid that it is able to adhere to vaginal epithelial cells, acting as a buffer in the vaginal secretions (Milani et al., 2000). Recently, a new benzoyl peroxide formulated polycarbophil/carbopol 934P hydrogel was shown to inhibit the growth of *G. vaginalis* with little or no effect on *Lactobacillus* spp. (Xu et al., 2013). Another agent that has been long used in the treatment of vaginal infections is boric acid (Van Slyke et al., 1981). Recently, Reichman and colleagues reported that the use of boric acid in combination with a nitroimidazole reduce the BV recurrence (Reichman et al., 2009), suggesting a potential impact on BV biofilms. However, this need to be further studied and *in vitro* biofilm experiments will elucidate the role of boric acid in BV prevention.

### Other anti-biofilm agents

An innovative approach to disrupt BV biofilms consists in the use of DNase which targets extracellular DNA. According to Hymes and colleagues, *G. vaginalis* biofilms contain extracellular DNA, which is essential to their structural integrity. In a series of *in vitro* studies, they showed that enzymatic disruption of extracellular DNA not only inhibited the formation of new biofilms but also destroyed the already formed ones (Hymes et al., 2013). In addition, DNase liberates bacteria from biofilms into the supernatant fractions and so potentiates the effect of metronidazole. Furthermore, using a murine model of vaginal colonization of *G. vaginalis*, these researchers also demonstrated that DNase treatment decreases the colonization density of *G. vaginalis.* Thus, DNase seems to be a promising therapeutic agent for BV either alone or in combination with antibiotics.

Another strategy involves the use of retrocyclin 101, a synthetic cyclic antimicrobial peptide with antiviral activity (Cole et al., 2007). Retrocyclin 101 and has been shown to inhibit the cytolytic activity of vaginolysin, a toxin produced by *G. vaginalis*, and to prevent *de novo* biofilm formation of this bacterial species (Hooven et al., 2012) while being well-tolerated by host tissues and by commensal vaginal bacteria (Eade et al., 2013).

## Conclusions and future directions

BV current approved therapies are not sufficient to deal with this multi-species biofilm-related vaginal disorder. Future, research should address biofilm communities with a particular emphasis on multi-species biofilms, a topic that only recently emerged (Castro and Cerca, 2015). By properly addressing the complex interactions established in multi-species biofilms, novel strategies will hopefully overcome the high recurrence and relapse rates associated with BV.

## Author contributions

DM prepared the first draft of the manuscript. JC prepared the first draft of the tables. NC, AO, and JO defined the content of the manuscript. All authors critically reviewed and approved the final version of the article.

## Funding

Research on BV biofilms in NC laboratory is supported by funding from the Fundação para a Ciência e a Tecnologia (FCT) strategic project of unit UID/BIO/04469/2013. DM and JC acknowledge the FCT fellowships SFRH/BD/87569/2012 and SFRH/BD/93963/2013 respectively. NC is an *Investigador FCT*.

### Conflict of interest statement

The authors declare that the research was conducted in the absence of any commercial or financial relationships that could be construed as a potential conflict of interest. LABFIT works in the development of plant-based therapheutics against vaginal infections; APO is co-president of Labfit.

## Abbreviations

BV: Bacterial Vaginosis
CDC: Centers for Disease Control and Prevention
LAE: Lauramide Arginine Ethyl Ester.
